# Biofeedback Systems for Gait Rehabilitation of Individuals with Lower-Limb Amputation: A Systematic Review

**DOI:** 10.3390/s20061628

**Published:** 2020-03-14

**Authors:** Rafael Escamilla-Nunez, Alexandria Michelini, Jan Andrysek

**Affiliations:** 1 Institute of Biomaterials and Biomedical Engineering, University of Toronto, Toronto, ON M4Y 1R5, Canada; rafael.escamilla@mail.utoronto.ca (R.E.-N.); a.michelini@mail.utoronto.ca (A.M.); 2 Bloorview Research Institute, Holland Bloorview Kids Rehabilitation Hospital, Toronto, ON M4G 1R8, Canada

**Keywords:** amputee, biofeedback, gait, locomotion, lower-limb amputation, prosthesis, real-time feedback, sensory feedback, rehabilitation, wearable systems

## Abstract

Individuals with lower-limb amputation often have gait deficits and diminished mobility function. Biofeedback systems have the potential to improve gait rehabilitation outcomes. Research on biofeedback has steadily increased in recent decades, representing the growing interest toward this topic. This systematic review highlights the methodological designs, main technical and clinical challenges, and evidence relating to the effectiveness of biofeedback systems for gait rehabilitation. This review provides insights for developing an effective, robust, and user-friendly wearable biofeedback system. The literature search was conducted on six databases and 31 full-text articles were included in this review. Most studies found biofeedback to be effective in improving gait. Biofeedback was most commonly concurrently provided and related to limb loading and symmetry ratios for stance or step time. Visual feedback was the most used modality, followed by auditory and haptic. Biofeedback must not be obtrusive and ideally provide a level of enjoyment to the user. Biofeedback appears to be most effective during the early stages of rehabilitation but presents some usability challenges when applied to the elderly. More research is needed on younger populations and higher amputation levels, understanding retention as well as the relationship between training intensity and performance.

## 1. Introduction

Lower-limb amputation (LLA) is associated with major rehabilitation challenges and lifelong mobility limitations. Limb loss not only hinders aspects of motor control, but it also reduces the sensory feedback information and proprioception that are associated with the peripheral nervous system [1,2,3,4]. As a result, individuals with LLA often walk slower and expend more energy than non-amputees [5]. They also exhibit atypical gait and loading patterns [5,6,7] that may be associated with long-term secondary health issues including chronic back pain and joint problems [8]. Moreover, poor balance and gait function in individuals with LLA can lead to the fear of falling and a greater incidence of falls [9,10,11,12], with more than half of ambulating adults with LLA falling at least once per year [11]. The consequences of these falls include injury and hospitalization [12,13], heightened fear of falling leading to prosthesis disuse [14,15], and the subsequent social withdrawal reducing their ability to recover from the trauma, both physically and psychologically [14,16].

Improving balance and gait is an important part of the rehabilitation process. Gait retraining, which is typically provided by a physiotherapist or prosthetist, includes the observation of gait deviations or atypical movement patterns and the provision of verbal cuing to promote corrections. However, these conventional methods have several limitations. The detection of gait deviations is limited to a subjective assessment of gross movement patterns [17]. Further, gait training sessions are limited in duration and frequency. Many health care systems are under-resourced despite the need for rehabilitation, making it challenging for clinics and rehabilitation hospitals to give adequate levels of therapy services [18]. Many patients face serious barriers (e.g., long travel times, school/work absences, etc.) accessing services, and, at best, only undertake unsupervised lower limb exercises at home [19,20,21].

Technology-driven approaches, such as virtual reality, therapy-focused videogames, and biofeedback (BFB) systems, are promising modalities for augmenting rehabilitation in the clinical facility and at home [22,23,24,25]. These technologies take advantage of motor learning and relearning strategies to accelerate the gait rehabilitation learning process. A major benefit of BFB systems, especially wearable systems, is the ability to provide real-time, continuous feedback to reinforce physiotherapy/prosthetist (PT) goals and good gait habits [26,27,28,29]. BFB approaches in rehabilitation consist of utilizing external sensors, such as inertial measurement units, goniometers, pressure sensors, force plates, and motion capture systems, to measure specific parameters relating to postural balance, gait kinetics, and kinematics [22,30,31,32,33]. Subsequently, real-time biomechanical information, which is captured by the external sensors, is communicated to the BFB users to alter their performance through some appropriate feedback modality (i.e., visual [34,35], auditory [36], haptic [25,26,37], or multimodal feedback [38,39]).

BFB approaches in rehabilitation have been studied in a variety of patient populations including stroke [37,40,41,42], Parkinson’s disease [43,44,45], cerebral palsy [46], vestibular deficits [47,48], diabetes [49], and upper-limb [50,51] and lower-limb amputees [31,52,53]. As well as in a variety of applications, including static and dynamic postural balance [54,55,56], walking [57,58,59], stairs management [60], obstacle avoidance [61], floor conditions identification [62], and sensory perception [31,53,63], to mention a few. In 2018, a mapping review [64] was published regarding the use of BFB for gait retraining. The review covered a variety of patient populations across 173 reviewed articles. The results showed that, during walking, the most common feedback modality, feedback gait parameter, and external sensory configuration were visual feedback, kinematic gait parameters, and pressure sensors fixed to the feet or on the feet insoles, respectively. In addition, it revealed that most of the studies (approximately 90%), tested BFB in a laboratory setting, and more than a half (i.e., 53%) of all studies performed a single intervention session. The relevance and extent of applicability to individuals with LLA remains uncertain despite these valuable new insights into BFB systems, since LLA was the subject of only eight of the 173 reviewed articles.

Similarly, earlier reviews relating to BFB have not directly focused on individuals with LLA, but more generally on a range of diagnoses. A 2010 systematic review [65] investigated the application of BFB in older adults with balance and mobility disorders [65]. Most of the reviewed articles involved post-stroke participants or community-dwelling elderly subjects. The three articles dealing with LLA showed that participants were able to adjust their weight on the injured leg during walking [66,67] and to improve sway and weight distribution during standing [68] when auditory feedback was provided about weight-bearing. Another systematic review [22] published in 2010 investigated the effectiveness of BFB systems for gait retraining of several related pathologies (e.g., cerebrovascular accident, hip fracture, etc.), including only one study on LLA. This review showed the potential of BFB systems to produce moderate to large short-term treatment effects and improvements as compared to conventional therapies when providing biomechanical information to BFB users [22]. It was further concluded that kinematic and spatiotemporal feedback gait parameters were the most targeted training interventions amongst stroke patients, while the kinetic feedback parameters were of primary interest for LLA [22].

While substantial research has been conducted toward the establishment of prosthetic BFB, to the authors’ knowledge, no study has attempted to consolidate this information to provide a comprehensive assessment of the state of the science, and the potential role of BFB in the treatment of LLA. In this regard, the main aim of the systematic review was to provide insights and recommendations towards the development of an effective, robust, and user-friendly wearable BFB system that can be integrated into existing prosthetic systems to assist individuals with LLA during the gait rehabilitation process. Specifically, this systematic review aimed to identify: (1) targeted populations and demographics within prosthetics, (2) targeted gait and biomechanical parameters, (3) the most common BFB system designs, including the measured biomechanical signals and feedback modalities, (4) the main technical and clinical limitations of current BFB systems, (5) main clinical evidence relating to BFB efficacy and effectiveness, and (6) future directions and applications of BFB systems for individuals with LLA.

## 2. Methods

The systematic review was conducted following the Preferred Reporting Items for Systematic Reviews and Meta-Analyses (PRISMA), see Supplementary Materials. The review protocol is register to PROSPERO, which is an international prospective register of systematic reviews (Ref: CRD42020142222).

### 2.1. Search Strategy

A literature search was performed on October 2019 on the following databases: Medline (1946–2019), Embase (1947–2019), PubMed (1971–2019), IEEE (1872–2019), Web of Science (1900–2019), and Scopus (2004–2019). These six databases were chosen as they cover most of the literature in the fields of engineering and medicine. Keywords “biofeedback”, “amputee”, and “gait” were matched with MeSH (medical subject headings) terms and subheadings when relevant. Truncations and wildcards were utilized to capture all forms of a root word. Table 1 shows an example of a search strategy performed in one of the databases (i.e., Medline). Minor modifications of the keyword terms were used in the different databases to expand the search results.

### 2.2. Inclusion and Exclusion Criteria

The article inclusion/exclusion criteria were divided into three main sections: (i) study population, (ii) biofeedback application, and (iii) publication type (Table 2).

### 2.3. Screening and Data Extraction

After the duplicates were removed, two independent reviewers screened the titles and abstracts of retrieved studies for relevance using the predefined eligibility criteria (Table 2) (R.E. & A.M.). The remaining studies received full-text assessments. Articles with titles and abstracts that did not provide enough information for the article screening process were fully reviewed. The data were extracted based on the study aims. Accordingly, the following aspects for data extraction were considered: (i) year of publication; (ii) BFB objective and application; (iii) characteristics of the sample population; (iv) BFB design (BFB modality, BFB device, feedback strategy, sensors/transducers); (v) testing conditions (clinical/laboratory settings or field-based studies and treadmill or overground walking); (vi) outcome measures (targeted gait parameters, physical, physiological, or any other parameters, including questionnaires); (vii) experimental protocol (information related to subject’s testing, such as number of sessions, duration, frequency, number of trials, follow-up interventions); and, (viii) key findings that were related to the efficacy and effectiveness of current BFB systems as gait rehabilitation tools for individuals with LLA. A third reviewer (J.A.) resolved the ambiguities or disagreements in the independent reviews of the articles (reviewers R.E. & A.M.). Additionally, the references of all included articles were scanned to identify other relevant studies that were missed in the original search.

### 2.4. Risk of Bias (Quality) Assessment

Most of the inclusions were peer-reviewed journal articles thus maintaining the quality of this systematic review and reducing the risk of publication bias. Two independent reviewers assessed all articles that met the inclusion criteria (R.E. & A.M.). In addition, a quality assessment was performed using a customized data extraction formula (Table 3). The approach was based on previous standardized methods [69,70,71] and reviews, and allows for data extraction that is relevant to the topic of interest [72,73]. For instance, Peters et al. [72] assessed 20 reviewed articles by using 19 appraisal questions as quality indicators. The appraisal questions were designed to collect information regarding the main research aims. Similarly, Ku et al. [73] utilized 14 appraisal questions to evaluate 23 articles that were related to balance control of individuals with LLA during quiet standing. The evaluation process in the current systematic review was adapted from these previously established appraisal criteria (Table 3) [69,70,71,72,73]. Accordingly, the score of each article provided a standardized measure for assessing the quality of research among the articles. Reviewers R.E. & A.M. independently applied the ratings and reviewer J.A. resolved disagreement.

## 3. Results

### 3.1. Search Results

**Figure 1:**
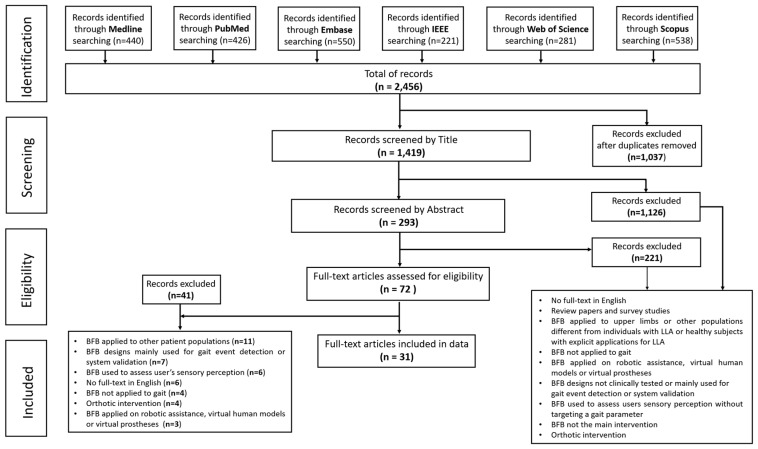
Preferred Reporting Items for Systematic Reviews and Meta-Analyses (PRISMA) flow diagram of the screening and data extraction process.

The initial search yielded 2456 studies (i.e., Medline 440, PubMed 426, Embase 550, IEEE 221, Web of Science 281 and Scopus 538). After the duplicates were removed, the title and abstract of 1419 articles were screened for potential relevance. Seventy-two (n=72) full-text articles were assessed for eligibility. Following the application of the eligibility criteria, thirty-one (n = 31) full-text studies were included in this systematic review. The flow diagram summarizes the overall review process (Figure 1). The most common reasons for the exclusion of articles during full-text assessment included: (1) BFB systems not being tested on individuals with LLA, (2) mainly used for gait event detection, and (3) used to assess user’s sensory perception (i.e., reaction time and subject’s accuracy in response to stimulation).

### 3.2. Quality of Reviewed Articles

Table 4 depicts the results of the criteria applied to assess the quality of the reviewed articles. Most articles were high quality and included complete information about the research objectives, study design, participants characteristics, BFB modality and application, BFB components, primary gait outcome measures, key findings supported by results, and conclusions. However, some articles presented limited information about experimental protocol and study limitations. Whereas, other studies presented limited or null information about statistical analyses and key findings supported by other literature. The results showed that 16 out of the 31 studies satisfied at least 85% of the criteria. Nine studies ranged from 70% to 85%, and six studies scored less than 70%. 

It should be noted that Lee et al. published five articles with similar methodology, data sets, and outcomes between the years of 2007–2013 [56,74,75,76,77].

### 3.3. Key Data Extracted from Reviewed Articles

BFB systems applied as a gait rehabilitation tool for prosthetic users has gained popularity over recent years, with most articles published from 2007 to 2019 (n = 21, 68%). The participants with transtibial amputation were most studied (n = 17, 55%) and the included studies had a median sample size of seven participants. Only two studies [34,66] compared BFB performance versus a control group of healthy subjects. Most of the studies included middle-aged (aged 30–59 years) and elderly (above 59 years) prosthetic users. Study participants had prosthetic experience ranging from one month to 53 years.

Only six studies presented detailed characteristics about the prosthetic components (i.e., prosthetic joint, foot, and socket) utilized to evaluate BFB [34,59,78,79,80,81]. During BFB testing, most of the participants wore their prescribed prosthesis (i.e., passive mechanical or microprocessor-controlled knee prostheses). In terms of BFB effectiveness and prosthetic components, the results showed that BFB systems were capable of improving the gait performance of individuals with LLA, regardless of the type of prosthetic components (i.e., passive mechanical knee or microprocessor-controlled knee or powered knee prostheses) [35,59,79,80,81,82].

FSRs (force sensitive resistors) sensors that were attached to the plantar surface of the prosthetic foot were the most frequently used transducer for measuring the targeted gait parameters. The most commonly targeted gait parameters were related to limb loading, ground reaction forces, and symmetry ratios for stance or step time. Visual feedback was the most used modality, followed by auditory and haptic. Haptic feedback has been most frequently used in recent studies. For instance, 10 out of 15 studies published from 2012 to 2019 utilized some type of haptic feedback (i.e., vibrotactile, electrotactile, electrocutaneous, or intraneural stimulation) when compared to two out of 16 studies during years 1975 to 2011.

Most studies assessed the performance of the BFB systems under laboratory conditions either walking on a treadmill or over ground. Most studies (above 50%) also performed only one gait training session in which BFB was delivered to the participants. Most studies compared subject’s gait performance with and without wearing the BFB system, walking at self-selected speed. Most of the studies did not report any follow-up sessions with the BFB system to test for retention. In addition, few studies evaluated changes on metabolic consumption [34,59], physical fatigue [59], and cognitive load or mental effort [58,59]. Most studies presented positive gait outcomes that were related to one or more physical and physiological parameters. However, six (n = 6) studies [34,36,57,83,84,85] reported mixed results, showing gait improvements for some participants and not others after BFB. None of the studies reported negative effects BFB on gait. Only two studies [84,85] reported non-persistent lasting effects and/or periods of retention after training. Table 5 details the key information that was retrieved from each article. 

## 4. Discussion

The primary aim of this systematic review was to consolidate published evidence that was related to the development and testing of BFB systems as a gait retraining tool for individuals with LLA. The quality of the identified studies (n = 31) was generally high, particularly in more recent years (i.e., since 1990). 

### 4.1. Sample Size

The average sample size across studies was 13 ± 3 non-amputee subjects, 7 ± 2 individuals with transtibial amputation, and 3 ± 1 individuals with transfemoral amputation. A few studies indicated that statistical tests could not be accurately performed and the findings cannot be extrapolated to larger populations due to their low sample size [56,58,96]. It was indicated that, although a small sample limits generalization of findings, a case study could provide pilot data and allow for exploratory research across a diverse population [97,98].

### 4.2. User Demographicss

The majority of lower limb amputees are over 50 years of age and most of the amputations are due to complications that are associated with vascular diseases [99,100]. The age of participants with LLA in this review ranged from young adults (as young as 19 years) to the elderly (aged 60 years and above). None of the studies focused on children and youth; a population that might benefit most due to a lifetime of prosthetic use and savviness for technology. There exist limitations that are associated with the transferability of findings between young and older populations. One reason for this is the differences in ambulation ability and the capacity to regain mobility function [100]. Elderly patients may require longer practice time, yet they typically suffer from lower physical endurance [17]. Further, with an older population come challenges with BFB usability. A recent study showed that, in contrast to healthy young adults, elderly healthy subjects were unable to utilize BFB information to reduce trunk sway while walking distracted [101]. Walking can be considered to be an unconscious (i.e., low cognitive) activity for healthy subjects [102]. However, for prosthetic users, walking often increases cognitive load and energy expenditure [103,104]. Prosthetic users usually depend on additional information (i.e., visual, auditory, and somatosensory) to ambulate safety [10].

Phantom limb pain affects many individuals with lower-limb amputation. It is manifested as sensations or pain from a body part that no longer exists [105]. Techniques, such as mirror therapy and BFB, have demonstrated the potential to reduce phantom limb pain in prosthetic users [59,86,105]. An important benefit of BFB systems when compared to mirror therapy approaches is that, in addition to reducing phantom pain, BFB systems can potentially improve the overall gait performance and prosthetic function of individuals with LLA [59,86]. Accordingly, participant characteristics (i.e., age, etiology, level of amputation, and prosthetic experience) should be considered in the development an effective and user-friendly BFB system, as these may dictate the most suitable BFB modalities (i.e., visual, auditory, or haptic feedback) and BFB strategies (i.e., control algorithms utilized to convey sensory feedback to BFB users). 

### 4.3. Level of Amputation

Transfemoral amputees typically demonstrate more severe gait deviations than transtibial amputees [106]. There is also increased energy cost, loss of mobility function and decrease in walking efficiency with higher levels of amputation [97]. However, transfemoral amputees are generally underrepresented in the BFB research. Additionally, the targeted gait parameters appear to be dependent on the specific amputee demographic. For instance, gait symmetry ratios, stance times, hip and knee flexion/extension angles, and trunk sway are more relevant for transfemoral than those for transtibial amputees [6,107]. One study found it difficult to recruit transtibial amputees with asymmetric gait and recommended recruiting transfemoral amputees [36]. On the other hand, the gait of transfemoral amputees and their ability to make gait adjustments is highly dependent on the function of the prosthesis, and particularly the prosthetic knee joint [56]. In one study, for example, because of the inability of the artificial knee-joint to actively adjust, the TF amputee could only vary gait speed with the healthy limb [108]. Hence, improvements in gait may require gait retraining, but also adjustments to the prosthetic setup, particularly with higher-level amputations. While this might become less of an issue with self-adjusting microprocessor knee joints, it will be imperative that the BFB and prosthetic control systems are designed to work symbiotically. It was also suggested that future research should determine how different amputee populations can benefit from different BFB modalities and different BFB strategies [28,56,88]. 

### 4.4. Prosthetic Experience and Time Since Amputation

The best practices in amputee rehabilitation encourage physiotherapy, prosthetic fitting, and training to be provided as soon as possible post amputation. One study involving established prosthetic users (>2 years post amputation) suggested that research should focus on how effective BFB is in the early stages of rehabilitation [56]. Others suggest that the best results occur with novice users, but that experiments should be done on expert prosthesis users to examine effectiveness [28]. To this point, one study found that newer, less established amputees were better able to adapt their gait patterns [36]. While early rehabilitation using BFB has been suggested for other populations, such as stroke, this approach presents certain challenges in amputee research and rehabilitation [109]. Issues, such as prosthetic fit and residuum healing can complicate experimentation on new amputees [97]. Overloading in the post-operation stage might result in tissue breakdown [90] and premature rehabilitation might affect the incision and cause healing issues [110]. BFB systems have been developed to improve the healing process in the early postoperative period by warning amputees applying excessive pressure on the residual limb [90]. Several studies had confounded results, since the patients were being provided conventional physiotherapy during the time of the BFB experiments; this is a potential issue when testing with recent amputees receiving standard care [81,86,97]. Similarly, to limit the confounding effects, one study withheld prosthetic alignment changes as the participants began to exhibit better gait patterns. However, this goes against standard care and might have contributed to poorer results in the study [81]. Based on these findings, BFB training should be provided as soon as the residuum is healed and volume stabilized, a satisfactory and stable prosthetic setup, alignment and fit have been achieved, and conventional physiotherapy treatments have concluded to exclusively assess the effectiveness of the BFB systems.

### 4.5. BFB Intervention (Experimental Protocol) 

None of the studies tested BFB under a randomized control trial (RCT). RCTs minimise the risk of confounding factors that might alter the results. For this reason, RCT studies are the golden standard for validating the effectiveness of an intervention. Over one-quarter of the studies reported using a single gait training session. It is important to allow for the user to have adequate training with the BFB system to enhance learning [64]. Training is important, as making errors drives motor learning [39]. Studies often do not report instructions given or training methods and research should be done to determine the best method of conducting the training sessions [111]. The effects of training intensity were mentioned in a few of the included papers and conclusions were mixed. One study concluded that outcomes are better with more intensive training [56], while others suggest gradually setting attainable targets [88]. Moreover, as in previous reviews, most studies did not report any follow-up sessions with the BFB system to test for retention [22,64]. Finally, the literature is unclear as to how BFB should be integrated into conventional physiotherapy. For example, systems, such as the one described by Redd et al., can be used with little specialized training and without the supervision of a physiotherapist/prosthetists [88]. It is likely that BFB might be most effective in combination with existing physiotherapy and gait training practices [112]. 

### 4.6. Treadmill vs. Overground Walking

Most studies used a treadmill during the experimental procedure. Nagano et al. has shown that temporal gait parameters, such as double stance time and swing time differ when walking on a treadmill compare than those walking overground [101]. Another study showed that walking on a treadmill reduces dorsiflexor and knee extensor moments [102] and increases hip extensor moments in the sagittal plane [102]. This suggests that BFB strategies utilized to alter gait symmetry might need to be modified, depending on treadmill or overground walking. Further, it has been shown that difficulty might arise when translating locomotor skills from treadmill training to overground walking [103].

### 4.7. BFB Parameter Measurements

Only some of the studies validated the BFB system’s accuracy in sensing the targeted gait parameter(s). For example, Isakov et al. validated their pressure sensing insoles with a force plate [66] and Yang et al. used a previously validated BFB system consisting of a force plate and motion analysis system [36,113]. The inaccuracy of a goniometer for knee angle measurements was a limitation in one study [87]. Another study noted a source of error in the algorithm to detect heel-strike and toe-off [88]. The accurate and reliable measurement of gait deviations is crucial to the success of wearable BFB systems, as erroneous feedback and false positives calling for corrections in gait can confuse and frustrate BFB users. Few studies mentioned time delays in their BFB system [30,87,114]. For instance, Crea et al. [30] and Liu et al. [114] reported delays less than or equal to 200ms due to wireless communication with the sensors embedded in the shoe-insoles. Consequently, delays were produced in the detection of the feedback stimulus. Accordingly, effective BFB systems must have low latency, especially when sensing and providing real-time feedback during dynamic activities, such as walking. 

### 4.8. BFB Modality

Visual feedback was the most common method provided to the participants, but there is some debate on the most appropriate and effective feedback modality. In one study, visual feedback was deemed the most intuitive modality that was based on user preference and usability [88]; however, the authors suggested that more work should be done to improve the usability/ease of use of vibrotactile and auditory feedback [88]. One study found that amputees and physiotherapists valued auditory over visual feedback and the participants adapted more easily to auditory feedback [56]. These preferences appear to be related to BFB design, testing, and safety aspects. BFB users might prefer or find more useful the BFB modality that provides more intuitive and relevant feedback information according to the gait parameter and task performed. For example, visual feedback is typically confined to specific locations (e.g., treadmill walking while watching a display under laboratory conditions). Safety issues relating to falling can arise during activities of daily living, for example, as prosthetic users walk and negotiate obstacles, such as curbs and stairs while watching a display or a smartphone. For this reason, auditory and haptic (e.g., vibrotactile) feedback may be more suitable for field or community-based systems [64]. A previous mapping review came to similar conclusions. It noted that, while visual feedback was most commonly used and studied, it might not be the most effective feedback modality for practical use [64]. Visual feedback is more appropriate for the perception of spatial information [115], while auditory and haptic feedback are better suited for the perception of temporal [115] and spatiotemporal information [39,115], respectively, according to the literature in motor learning. Accordingly, BFB systems need to appropriately align feedback modalities and strategies with measured gait parameters. For instance, in terms of vibrotactile feedback, diverse feedback strategies (i.e., a combination of vibration patterns—vibration levels and activation sequences—including different locations and number of vibrating motors) have been utilized to improve gait performance [28,36,40,58,88,116]. However, a systematic comparison of the current implemented feedback strategies is missing to explore which strategy might produce greater positive gait outcomes on individuals with LLA. 

Of the reviewed studies, six (n = 6) provided multimodal feedback [58,74,75,76,88]. Some researchers have suggested that multimodal feedback reduces cognitive load and can enhance motor learning [39,58,83]. For instance, Crea et al. evaluated the cognitive load of a visual-vibrotactile BFB system by adding a secondary cognitive task (i.e., serial subtraction) during walking with vibrotactile feedback [58]. The results showed that gait symmetry was improved without significant increases on cognitive load in the presence of feedback walking [58]. In another study, Pagel et al. showed that, when cognitive load increases, the imbalance between intact and prosthetic limb becomes more pronounced [84]. Cognitive impairment appears to be more common in individuals with LLA than in the general population—this is linked to difficulties with regaining mobility and independence after amputation [117]. Thus, if the feedback is too cognitively taxing, it might be counterproductive and more difficult for amputees to process the information, potentially even resulting in worsened gait and mobility performance [112]. For instance, Fernie et al. [85] designed an auditory BFB system to maintain knee extension through the stance phase; however, the BFB system was found to alter knee flexion instead. Chow et al. [90] originally designed an auditory BFB to encourage participants to increase the loading of the residual limb, but in fact the audio BFB prevented adequate loading of the residual limb. 

### 4.9. Feedback Strategies

Feedback strategies mainly utilized baseline (no feedback) conditions to obtain an initial value of the specific gait parameters. Most of the studies set a gait target threshold for participants prior to BFB testing. Few studies set these thresholds based on the feedback provided by a physiotherapist, who assessed the participant’s walking ability to personalize the BFB treatment or provided verbal cues prior or during the early stages of BFB gait retraining [17,34,36,81,85,94]. Feedback can be provided in real-time (concurrent feedback) or after the trial has finished (terminal feedback) [112]. All the studies applied concurrent feedback, and none provided terminal feedback. A recent review [112] showed that concurrent feedback produces the best short-term results, while terminal feedback produces the best long-term results [112]. Therefore, an effective training strategy could be to provide concurrent BFB and terminal feedback from the physiotherapist during training sessions. 

### 4.10. Other BFB design and Application Considerations

When designing a BFB system, it is important that the BFB strategy is non-obtrusive and enjoyable for the user. Program adherence has been linked to program enjoyment [97]. This will ensure that the BFB systems will be used in the long-term. Motivation for walking is a predictive factor for successful rehabilitation of amputees [118]. If a wearable daily use system is not the goal for the researcher, virtual reality (VR) systems may be a good option to motivate and encourage program adherence, since users might perceive to have more interaction with the system. For balance, the Wii-Fit has been shown to improve the balance and gait in older adults [97], children and adolescents with amputation [119], as well as individuals with Alzheimer’s and Parkinson’s disease [120]. Although the Wii-Fit is no longer commercially available, other VR options have been used for rehabilitation purposes, including the CAREN system and C-Mill (both from Motekforce Link, Amsterdam). The two included studies used VR environments, specifically the CAREN system [34,81]. Virtual reality might be the best option for BFB because program adherence is important [97]. Alternately, if BFB systems are envisioned as wearable systems that are built into prostheses that provide as needed feedback during activities of daily living, they must do so unobtrusively and seamlessly. However, to date, only two studies tested their systems in the field [59,86]. Moreover, retention and fading, which are important considerations for the continuous use of feedback systems, are not well understood and require further attention [64].

### 4.11. Limitations of the Systematic Review

It was not possible to conduct a meta-analysis on the data due to the wide range of target gait parameters, outcome measures, and methods used. Further, six databases as well as the references from included studies were used to search for articles. Articles that were not written in English were not included.

## 5. Conclusions

Although most individuals with amputation are older, there is a lack of research on technology-based feedback for a younger or paediatric population. Further, the older population might have difficulty with the usability and response times of BFB systems. Different amputation levels may benefit from different feedback strategies and/or target parameters; therefore, it is important to investigate the effect on gait of different feedback strategies to ensure that the target gait parameter and the sensory information are appropriate for the target population. BFB training should be provided as soon as possible in the rehabilitation stage, but the training should not start until the stabilization of the early stages of the rehabilitation process. Auditory and vibrotactile feedback are more wearable and different population ages may respond to feedback differently, and it is important to align feedback modality and feedback strategies appropriately with the measured gait parameter(s). The relationship between training intensity and performance is unknown and future work should be conducted to investigate a possible correlation.

In terms of developing an effective, robust, and user-friendly wearable biofeedback system to improve the gait of individuals with LLA, the following aspects should be considered: (1) target gait parameters should be clinically relevant for the targeted population. For instance, gait symmetry ratios, stance times, hip and knee flexion/extension angles, and trunk sway are more relevant for transfemoral than those for transtibial amputees; (2) BFB modalities (i.e., visual, auditory, haptic, and multimodal feedback) should take into consideration usage conditions (i.e., laboratory, clinical, or home-care settings), including user’s age, level of amputation, and prosthetic experience; (3) feedback information (i.e., BFB strategies) should be easy to perceive, discriminate, and utilize by BFB users, allowing for them to transfer feedback information with low cognitive demand; (4) wearable sensors, such as pressure sensors, load cells, electrogoniometers, etc. should be fully integrated into BFB systems to improve wearability. In addition, accelerometers, gyroscopes, or inertial measurement units (IMU sensors) are encouraged to be used for gait event detection, which might improve accuracy and portability of the BFB systems; and, (5) program adherence and program enjoyment should be sought to ensure the long-term use of BFB systems. Effective BFB systems might be achieved by designing a goal-oriented experimental intervention and by considering the previously mentioned points regarding BFB design.

## Figures and Tables

**Table 1:** Keyword search strategy employed in Medline database.

Biofeedback		Gait		Amputation
biofeedback.mp. OR feedback.kf,tw. OR (feedback adj3 sensory).mp. OR (wearable adj3 feedback).mp. OR (biomechanical adj3 feedback).mp. OR prosthesis design.tw,kf.	**AND**	gait.mp. OR walk*.mp.	**AND**	amput*.mp. OR prosthe*.tw,kf. OR (lower adj3 limb*).mp. OR (artificial adj3 limb*).mp. OR (artificial adj3 leg*).mp. OR (prosthe* adj3 leg*).mp. OR (prosthe* adj3 limb*).mp. OR knee prosthe*.mp. OR (prosthe* adj3 joint*).mp. OR (artificial adj3 joint*).mp. OR (lower adj3 extrtemit*).tw,kf. OR amputation/or disarticulation/or hemipelvectomy/ OR disarticulation.mp. OR hemipelvectomy.mp. OR (partial* adj3 amput*).mp.

kf: keyword heading word; tw: text word; mp: multi-purpose field search (Title, Original Title, Abstract, and Subject Heading, among others depending on the database).

**Table 2:** Framework for Inclusion/Exclusion of Eligible Studies.

Section	Criteria	
	Inclusion	Exclusion
**Study Population**	Individuals with lower-limb amputation Able-bodied subjects with explicit future application to lower-limb amputation population	Individuals with upper-limb amputation Other populations
**Biofeedback application**	Gait or walking applications	BFB applied to any activity different from walking (e.g., balance, running, golfing) BFB applied for robotic assistance (e.g., exoskeletons) or computer simulations (e.g., virtual human models or virtual prostheses) BFB used exclusively to assess user sensory perception of participants without targeting a gait parameter while walking BFB designs not clinically tested or mainly used for gait event detection or system validation BFB does not explicitly inform the user of errors that they are exhibiting (e.g., mirror therapy studies, studies where users observe videos of their own gait patterns without having deviations pointed out to the them)
**Publication Type**	Peer reviewed journal articles Peer review conference proceedings Studies published in English Full-text articles	Literature reviews Survey studies

**Table 3:** Criteria for Quality Assessment **.

Question
1. Were the research objectives of the study clearly stated? 2. Was the study design clearly described? 3. Were the subject’s characteristics and details clearly provided? 4. Was biofeedback modality (e.g., visual, auditory, haptic) and application clearly stated? 5. Was equipment design and setup clearly described? 6. Was the experimental protocol/subject intervention clearly defined? 7. Were the methods for statistical analysis clearly described? 8. Were the main outcomes measures clearly stated? 9. Were key findings supported by the results? 10. Were limitations of the study clearly described? 11. Were key findings supported by other literature? 12. Were conclusions drawn from the study clearly stated?

^**^ Questions were scored as follows: 2 = Yes; 1 = Limited detail; 0 = No.

**Table 4:** Quality analysis results from reviewed articles.

Study, Author	Year	Question												Total Score	Overall Percentage
		*1*	*2*	*3*	*4*	*5*	*6*	*7*	*8*	*9*	*10*	*11*	*12*		
[59] Petrini et al.	2019	1	2	2	2	2	2	2	2	2	2	2	1	22/24	92
[57] Fiedler et al.	2019	2	2	2	2	2	2	0	1	2	2	1	2	23/24	96
[80] Petrini et al.	2019	2	2	1	2	2	2	2	2	2	2	2	1	22/24	92
[35] Brandt et al.	2019	2	2	2	2	2	1	2	2	2	2	2	2	23/24	96
[86] Dietrich et al.	2018	2	2	2	2	2	1	2	2	2	2	2	2	23/24	96
[34] Esposito et al.	2017	2	2	1	2	2	2	2	2	2	2	2	2	23/24	96
[87] Maldonado et al.	2017	2	2	1	2	2	2	1	2	2	1	0	2	19/24	79
[58] Crea et al.	2017	2	2	1	2	2	2	2	2	2	2	2	2	23/24	96
[28] Plauche et al.	2016	2	2	2	2	2	2	0	2	2	2	2	1	21/24	88
[84] Pagel et al.	2016	2	2	2	2	2	1	2	2	2	2	2	2	23/24	96
[82] Huang et al.	2016	2	2	2	2	2	2	2	2	2	2	1	2	23/24	96
[30] Crea et al.	2015	2	2	1	2	2	2	2	2	2	2	2	2	23/24	96
[77] Lee et al.	2013	2	2	2	2	2	1	0	2	1	1	1	2	18/24	75
[88] Redd et al.	2012	2	2	1	2	2	2	2	2	2	2	2	2	23/24	96
[36] Yang et al.	2012	2	2	2	2	2	1	1	2	2	2	0	2	20/24	83
[81] Darter et al.	2011	2	2	2	2	2	2	0	2	2	2	2	2	22/24	92
[76] Lee et al.	2010	2	2	2	2	2	1	0	2	1	1	1	2	18/24	75
[75] Lee et al.	2009	2	2	2	2	2	1	0	2	1	1	1	2	18/24	75
[74] Lee et al.	2008	2	2	2	2	2	1	0	2	1	1	1	2	18/24	75
[56] Lee et al.	2007	2	2	2	2	2	1	0	2	1	1	1	2	18/24	75
[66] Isakov et al.	2007	2	2	2	1	2	2	2	2	2	0	0	2	19/24	79
[89] Davis et al.	2004	2	2	2	1	1	2	2	2	2	2	2	2	22/24	92
[90] Chow et al.	2000	2	2	2	2	2	2	2	2	2	1	1	2	22/24	92
[91] Dingwell et al.	1996	2	2	1	2	2	2	2	2	2	2	2	2	23/24	96
[83] Sabolich et al.	1994	2	2	2	2	2	2	1	2	2	1	0	2	20/24	83
[17] Flowers et al.	1986	2	1	1	2	2	0	0	1	2	2	0	2	15/24	63
[92] Clippinger et al.	1982	1	1	2	2	2	2	0	1	1	0	1	1	14/24	58
[93] Gapsis et al.	1982	2	1	2	2	2	1	1	1	1	1	1	1	16/24	67
[85] Fernie et al.	1978	1	1	1	1	1	1	0	1	1	1	0	1	10/24	42
[94] Zimnicki et al.	1976	1	1	1	2	2	2	0	1	1	1	0	1	13/24	54
[95] Warren et al.	1975	2	1	1	1	1	2	0	1	1	1	1	1	13/24	54

Questions were scored as follows: 2 = Yes; 1 = Limited detail; 0 = No. Questions were related to the description or justification of (1) Objectives; (2) Study Design; (3) Participant characteristics; (4 and 5) BFB system; (6) Experimental protocol; (7) Statistics; (8) Main Outcome Measures; (9 & 11) Key Findings; (10) Limitations; and, (12) Conclusions.

**Table 5:** Key data extracted from the reviewed articles (n = 31).

Study Characteristics	Participant’s Characteristics	Biofeedback (BFB) Design	Testing Conditions	Outcome Measures			Intervention Protocol Summary	Key Findings
				Gait Parameters	Physical, Physiological and Other Parameters	Questionnaire		
[59] **Petrini et al. 2019** Real-time intraneural stimulation to restore sensory feedback of transfemoral amputees	2 TF **Cause**: trauma **Age**: 49 yrs, 35 yrs **PE**: N/D **TSA**: 3 yrs, 12 yrs	**FM**: Intraneural stimulation (touch, pressure, or vibration) **FD**: Surgical implanted electrodes **FS**: Concurrent **S/T**: Insole pressure sensors, knee encoder	Lab & Field, Treadmill & Overground	Heel-strike, flat foot, toe-off, knee angle, walking speed	Metabolic consumption (VO_2_), mental effort, phantom limb pain	Neuropathic Pain Symptom Inventory (NPSI), Visual Analog Scale (VAS)	*Walking speed and mental effort:* 6 min outdoor (sand) walking x 2 sessions per condition (with/without feedback + dual task), *Metabolic cost:* Indoors: (i) 15 min treadmill walking with increasing speed, (ii) outdoors (grass): 3 min baseline x 6 min walking at SS speed	Walking speed and self-reported confidence increased. Mental and physical fatigue decreased, including reduced phantom limb pain with feedback
[57] **Fiedler et al. 2019** Mobile visual feedback system for gait rehabilitation in everyday-life environment	1 TT **Cause**: N/D **Age**: 61 yrs **PE**: 12 yrs **TSA**: N/D	**FM**: Visual **FD**: Smart glasses **FS**: Concurrent **S/T**: Load cell	Lab, Overground	Stance/step ratio, gait symmetry index	N/A	N/A	30 m walking (repeatedly) at SS speed within 1-hr	A strong correlation found between stance/step ratio (the feedback variable) and gait symmetry index
[80] **Petrini et al. 2019** Real-time tactile and proprioceptive feedback to increase prosthesis embodiment and to improve mobility of transfemoral amputees	3 TF **Cause**: trauma **Age**: N/D **PE**: N/D **TSA**: 3 yrs, 7 yrs, 12 yrs	**FM**: Intraneural stimulation (touch, pressure, or vibration) **FD**: Surgical implanted electrodes **FS**: Concurrent **S/T**: Insole pressure sensors, knee encoder	Lab, Overground	Heel-strike, flat foot, toe-off, knee angle, walking speed	Error walking on a line (walking agility), proprioceptive displacement, cognitive load (dual-task paradigm)	Embodiment questionnaire	Nine 5 m walking trials with/without feedback over a straight line (one foot after the other without stepping outside the line)	Improved mobility, ease of cognitive effort, and increased embodiment of prosthesis with feedback
[35] **Brandt et al. 2019** Visual feedback to increase stance time on the prosthetic limb. Compare powered versus passive knee prostheses	5 TF or knee disarticulation **Cause**: trauma/cancer/congenital **Age**: 19–59 yrs **PE**: 6 mos.–6 yrs **TSA**: 4–47 yrs	**FM**: Visual **FD**: Computer monitor **FS**: Concurrent **S/T**: Instrumented treadmill (dual belt) with force plates, motion capture system	Lab, Treadmill	Stance time, swing time, stance time asymmetry, peak anterior-posterior ground reaction forces, peak anterior propulsive asymmetry	N/A	Likert scale (perceived difficulty)	Twelve 1.5 min walking trials at SS speed with 2 min of rest between trials over 3 sessions of 3 h each. Fitting and training provided during prior sessions.	Stance time symmetry and peak propulsion symmetry significantly improved with both prosthesis by increasing prosthetic stance time via feedback
[86] **Dietrich et al. 2018** Assess whether prostheses with somatosensory feedback can reduce phantom limb pain and increase ambulation	14 TT **Cause**: trauma/embolism **Age**: 27–76 yrs (56.3 ± 11.6 yrs) **PE**: N/D **TSA**: 1–54 yrs	**FM**: Electrocutaneous **FD**: Electrodes **FS**: Concurrent **S/T**: Insole pressure sensors	Field, Overground	Stance time	Walking distance, walking speed, phantom limb pain	Likert scale (discrimination performance), Houghton Score Questionnaire (HSQ), Locomotor Capability Index (LCI), Trinity Amputation and Experience Scales (TAPES), Amputee Body Image Scale (ABIS), Pain questionnaires, and pain daily reports	10 days of training (walking at level ground and uneven terrains) over 2 weeks, 2 sessions per day, 2 h per session with 30–60 min of rest between daily sessions.	Reduction of phantom limb pain, larger walking distances, stable walking and better posture control on uneven ground with feedback
[34] **Esposito et al. 2017** Assess whether visual feedback can reduce center of mass sway and metabolic consumption during gait retraining	*Study group:* 8 TT **Cause**: trauma **Age**: 32.9 ± 5.7 yrs **PE**: 29 ± 38 mos. **TSA**: N/D *Control group:* 8 H **Cause**: N/A **Age**: 29.4 ± 3.8 yrs **PE**: N/A **TSA**: N/A	**FM**: Visual (virtual reality) **FD**: CAREN (Computer Assisted Rehabilitation Environment) **FS**: Concurrent **S/T**: Bipolar surface electrodes, motion capture system	Lab, Treadmill	Center of mass sway	Metabolic rate (VO_2_), heart rate, thigh muscle activation magnitudes and duration, quadriceps and hamstrings muscle activity	N/A	*Baseline*: 10 min in seated position (VO_2_ baseline). *Acclimation*: 4 min practice receiving visual feedback and verbal cues (PT). *Data collection*: 8 min walking (with/without visual feedback) at standardized speed	Visual feedback decreased center of mass sway and quadriceps activity. Thigh muscle co-contraction indices unchanged. Metabolic rate was not significantly affected by feedback
[87] **Maldonado et al. 2017** BFB system developed as a training tool to sense perturbations to perform corrective actions to avoid falls	2 TT **Cause**: N/D **Age**: 49 yrs, 67 yrs **PE**: N/D **TSA**: N/D	**FM**: Vibrotactile **FD**: Vibrating motors, solenoid **FS**: Concurrent **S/T**: Electrogoniometer	Lab, Overground	Knee angle	Reaction times, subject’s response to stimulus	N/A	Six 1 h to 2 h training sessions over 3 weeks, receiving only vibrotactile feedback. One 2 h session, vibrotactile and solenoid feedback (retention and transfer test)	Subjects performed the corrective movement in response to feedback. No conclusive results for retention and transfer tests.
[58] **Crea et al. 2017** BFB system developed to improve temporal gait symmetry of elderly transfemoral amputees	3 TF **Cause**: N/D **Age**: > 60 yrs **PE**: N/D **TSA**: > 1 year	**FM**: Vibrotactile, Visual **FD**: Vibrating motors, display screen **FS**: Concurrent **S/T**: Pressure-sensitive insoles	Lab, Treadmill	Stance time, symmetry index, cadence	Heart rate, breathing rate, skin temperature, skin conductance, cognitive load	National Aeronautics and Space Administration Task Load Index (NASA-TLX34), System Usability Scale (SUS)	Within a week: Pre- and Post-training, 1 session each (only vibrotactile). 3 sessions training (vibrotactile + visual feedback). Follow-up a week after (only vibrotactile)	Feedback improved symmetry index and lower cadence promoting longer strides. Cognitive load did not increase with feedback. No signs of negative psychophysiological effects.
[28] **Plauche et al. 2016** Develop a BFB system to asses gait performance under different vibrotactile feedback strategies on able-bodied subjects walking with a prosthetic adaptor	9 H (above-knee prosthetic adaptor) **Cause**: N/A **Age**: 25.6 ± 2 yrs **PE**: N/A **TSA**: N/A	**FM**: Vibrotactile **FD**: Vibrating motors **FS**: Concurrent **S/T**: Force sensing resistors (FSRs) sensors	Lab, Treadmill	Stride length step width, trunk sway, including their variabilities	N/A	Likert scale (feedback strategies)	Walking 30 s at SS speed on a treadmill (10 trials per condition) with/without feedback and with/without prosthesis adaptor	Improved gait stability as the variability of stride length, step width and trunk sway decreased.
[84] **Pagel et al. 2016** Develop a BFB system to improve gait symmetry by providing feedback from foot center of pressure and knee flexion angle	3 TF **Cause**: trauma/cancer **Age**: 21 yrs, 54 yrs, 73 yrs **PE**: 1 yrs, 36 yrs, 53 yrs **TSA**: 1 yrs, 52 yrs, 53 yrs	**FM**: Electrotactile **FD**: Electrodes **FS**: Concurrent **S/T**: Force/moment sensor, goniometer-gyroscope sensor	Lab, Treadmill	Stance time, step length, stance time ratio, step length ratio, ground reaction forces, center of pressure (CoP), knee flexion angle	N/A	User’s feedback experience questionnaire	2 min walking per condition (no feedback, CoP feedback, and knee angle feedback), SS speed	No persistent positive effect but improved step length for one participant. Subjects felt more benefited from knee angle feedback than CoP feedback.
[82] **Huang et al. 2016** Utilize visual feedback to alter prosthetic ankle performance while using a powered prosthesis with myoelectric controlled	5 TT **Cause**: trauma/cancer **Age**: 23–70 yrs (55.4 ± 18.6 yrs) **PE**: N/D **TSA**: 4–44 yrs (22.6 ± 19 yrs)	**FM**: Visual **FD**: Computer monitor **FS**: Concurrent **S/T**: Motion capture system, force plates, electromyography (EMG) sensors	Lab, Treadmill	Peak ankle power, total ankle work, positive ankle work, negative ankle work	Residual limb muscle activation patterns	N/A	5 min to 10 min walking trial with prescribed and powered prosthesis with/without feedback, speed 1.0 m/s. An average of 3.5 h of training in total over 2 months.	Adapted muscle activation patterns due to visual feedback. Increased peak ankle power and positive ankle work.
[30] **Crea et al. 2015** BFB system to provide vibrotactile feedback during gait-phase transitions	10 H **Cause**: N/A **Age**: 27 ± 1.8 yrs **PE**: N/A **TSA**: N/A	**FM**: Vibrotactile **FD**: Vibrating motors **FS**: Concurrent **S/T**: Pressure-sensitive insoles	Lab, Treadmill	Stance time, swing time, step cadence, vertical ground reaction force, center of pressure	N/A	Self-assessment questionnaire (cognitive effort)	6 min walking per condition (missing stimuli, delay stimuli: 200 s & 500 s, and wrong stimuli).	Accuracy in stimuli detection decreased if delay increased. Good usability, feedback is readily perceived by participants.
[77] **Lee et al. 2013** Evaluate a BFB system using subsensory stimulation and visual-auditory feedback to improve postural sway and dynamic weight shifting stability	7 TT **Cause**: N/D **Age**: 24–60 yrs (38.8 ± 14.08 yrs) **PE**: > 2 yrs (8.5 ± 6.12 yrs) **TSA**: N/D	**FM**: Auditory, Visual **FD**: PC speaker, computer monitor **FS**: Concurrent **S/T**: Force sensing resistors (FSRs) sensors	Lab, Treadmill	Double support time symmetry index, constant time step number index, single support time symmetry index, gait phase time ratio index	Heart rate	N/A	20 min each test session (5 min warm up, 10 min training and 5 min cool down). Walking speed increased each minute as tolerated (starting at SS speed)	Improvement in weight shifting stability indices. Most subjects easily adapted to auditory rather than visual biofeedback.
[88] **Redd et al. 2012** Assess the ability of a BFB system to alter gait symmetry under visual, auditory and vibrotactile feedback	12 H **Cause**: N/A **Age**: N/D **PE**: N/A **TSA**: N/A	**FM**: Auditory, Vibrotactile, Visual **FD**: Smartphone **FS**: Concurrent **S/T**: Force sensing resistors (FSRs) sensors	Lab, Overground	Stance time symmetry ratio	N/A	Usability survey	Six 200 ft walking trials (one trial per feedback modality and 3 trials with the preferred feedback modality)	BFB altered gait of user without supervision from a specialist. Visual was the preferred feedback modality.
[36] **Yang et al. 2012** Evaluate the performance of a BFB device to improve gait symmetry of prosthetic users	3 TT **Cause**: infection/embolism **Age**: 22–65 yrs (49.7 ± 19.6 yrs) **PE**: N/D **TSA**: 7 mos.–5.5 yrs	**FM**: Auditory **FD**: BFB buzzer **FS**: Concurrent **S/T**: Force sensing resistors (FSRs) sensors, motion capture system, force plates	Lab, N/D	Stance time, symmetry ratio, trunk sway	N/A	N/A	Pre-test 1 week before, six 30 min training, post-test 1 week after. PT set trial duration (avg. 30s–240s) and feedback thresholds.	2 of 3 subjects improved symmetry ratio and trunk sway
[81] **Darter et al. 2011** Assess biomechanical and physiological effects of gait training using virtual reality	1 TF **Cause**: trauma **Age**: 24 yrs **PE**: 2 yrs **TSA**: N/D	**FM**: Visual (virtual reality) **FD**: CAREN (Computer Assisted Rehabilitation Environment) **FS**: Concurrent, verbal cues (PT) **S/T**: Motion capture system, force plates	Lab, Treadmill & Overground	Frontal-plane trunk motion, frontal plane hip, pelvis and trunk angles, walking speed, step length, stance time, step width	VO_2_ consumption	N/A	Twelve 30 min walking sessions within 3 weeks. Follow-up: 3 weeks after training. PT involved during first BFB sessions	Training effective in improving frontal plane hip, pelvis and trunk motion, with decreases in oxygen consumption during overground walking. Retention found at 3 weeks after training
[76] **Lee et al. 2010** Asses a BFB system using subsensory stimulation and visual-auditory feedback to improve postural sway and dynamic weight shifting stability	7 TT **Cause**: N/D **Age**: 24–60 yrs (38.8 ± 14.08 yrs) **PE** > 2 yrs (8.5 ± 6.12 yrs) **TSA**: N/D	**FM**: Auditory, Visual **FD**: PC speaker, computer monitor **FS**: Concurrent **S/T**: Force sensing resistors (FSRs) sensors	Lab, Treadmill	Double support time symmetry index, constant time step number index, single support time symmetry index, gait phase time ratio index	Heart rate	N/A	20 min each test session (5 min warm up, 10 min training and 5 min cool down). Walking speed increased each minute as tolerated (starting at SS speed)	Improvement in weight shifting stability indices. Most subjects easily adapted to auditory rather than visual feedback
[75] **Lee et al. 2009** Assess a BFB system using subsensory stimulation and visual-auditory feedback to improve postural sway and dynamic weight shifting stability	7 TT **Cause**: N/D **Age**: 24–60 yrs (38.8 ± 14.08 yrs) **PE**: > 2 yrs (8.5 ± 6.12 yrs) **TSA**: N/D	**FM**: Auditory, Visual **FD**: PC speaker, computer monitor **FS**: Concurrent **S/T**: Force sensing resistors (FSRs) sensors	Lab, Treadmill	Double support time symmetry index, constant time step number index, single support time symmetry index, gait phase time ratio index	Heart rate	N/A	20 min each test session (5 min warm up, 10 min training and 5 min cool down).Walking speed increased each minute as tolerated (starting at SS speed)	Improvement in weight shifting stability indices. Most subjects easily adapted to auditory rather than visual feedback
[74] **Lee et al. 2008** Evaluate a computerized foot pressure BFB system using subsensory electrical stimulation and visual-auditory feedback to improve gait and balance of transtibial amputees	5 TT **Cause**: N/D **Age**: 24–48 yrs (37.4 ± 11.57 yrs) **PE**: >2 yrs **TSA**: N/D	**FM**: Auditory, Visual **FD**: PC speaker, computer monitor **FS**: Concurrent **S/T**: Force sensing resistors (FSRs) sensors	Lab, Treadmill	Double support time index, constant time cadence index, single support time index, stance/swing phase index	Heart rate	N/A	20 min each test session (5 min warm up, 10 min training and 5 min cool down). Walking speed increased each minute as tolerated (starting at SS speed)	Improvement in all dynamic gait performance indices. Most subjects easily adapted to auditory rather than visual feedback
[56] **Lee et al. 2007** Assess a computerized foot pressure BFB system using low-level electrical stimulation and visual-auditory feedback to improve gait and balance	7 TT **Cause**: N/D **Age**: 24–60 yrs (38.8 ± 14.08 yrs) **PE**: > 2 yrs (8.5 ± 6.12 yrs) **TSA**: N/D	**FM**: Auditory, Visual **FD**: PC speaker, computer monitor **FS**: Concurrent **S/T**: Force sensing resistors (FSRs) sensors	Lab, Treadmill	Double support period, constant time cadence, single support period, stance/swing ratio	N/A	N/A	20 min each test session (5 min warm up, 10 min training and 5 min cool down). Walking speed increased each minute as tolerated (starting at SS speed)	Improvement in all dynamic gait performance measures. Most subjects easily adapted to auditory rather than visual feedback
[66] **Isakov et al. 2007** Evaluate the effectiveness of a BFB system compare to traditional training (control group) to improve full weight-bearing of lower-limb amputees	42 LLA (TF, TT, hip and knee replacement, femoral neck fracture), (n = 22 study, n = 20 control group) **Cause**: N/D **Age**: avg. 62 yrs (study), 66 yrs (control) **PE**: N/D **TSA**: N/D	**FM**: Auditory (study), Verbal cues (control) **FD**: SmartSte™ (audio) **FS**: Concurrent (study), Physiotherapy (control) **S/T**: Pressure sensors (study)	N/D, N/D	Prosthetic weight-bearing	N/A	N/A	Both groups: 10 m walking at SS speed. Four 30 min sessions within 14 days.	Weight-bearing on the prosthetic limb was statistically significant increased while using BFB
[89] **Davis et al. 2004** Evaluate whether a BFB system is capable to reduce oxygen consumption by improving gait symmetry of lower-limb amputees	11 TF/TT **Cause**: trauma/diabetes **Age**: 36–58 yrs **PE**: N/D **TSA**: N/D	**FM**: Visual **FD**: Computer monitor **FS**: Concurrent **S/T**: Instrumented treadmill with force plates	Lab, Treadmill	Stance/swing ratios, foot propulsive forces, shear foot forces	Heart rate, VO_2_ consumption, tidal volume	N/A	Five 4 min tests with/without feedback per each target gait parameter (stance/swing ratio, foot propulsive forces, and shear foot forces)	Real-time visual feedback results in immediate symmetry improvements. Significant reductions in heart rate and oxygen consumption with feedback
[90] **Chow et al. 2000** Evaluate the effects of BFB on weight-bearing patterns of TT amputees at early postoperative period	6 TT **Cause**: diabetes/peripheral vascular disease **Age**: 66–78 yrs **PE**: N/D **TSA**: N/D	**FM**: Auditory **FD**: BFB buzzer **FS**: Concurrent **S/T**: Load-monitoring device (pair of single-axis strain gauges)	Lab, Overground	Prosthetic weight-bearing	N/A	N/A	4 randomized walking trials (5m length) with/without feedback over 5 sessions at SS speed	Feedback prevents overloading of the residual limb beyond the pre-set load target
[91] **Dingwell et al. 1996** Reduce gait asymmetries of TT amputees via real-time visual feedback	6 H **Age**: 33–54 yrs (avg. 42.7 yrs); 6 TT **Cause**: trauma/cancer/peripheral vascular disease **Age**: 31–69 yrs (avg. 41.7 yrs) **PE**: 6 mos.–21 yrs (avg. 6 yrs) **TSA**: N/D	**FM**: Visual **FD**: Computer monitor **FS**: Concurrent **S/T**: Instrumented treadmill with force plates	Lab, Treadmill	Centre of pressure (CoP), stance time (%), push off forces, symmetry index, single support time	N/A	N/A	4 min of acclimation (no feedback), 5 min of training with each feedback parameter (CoP, stance time percentage, and symmetry index), SS speed	Asymmetrical gait patterns were significantly reduced after providing visual feedback
[83] **Sabolich et al. 1994** Improve balance and gait by restoring sensory perception at the residual limb/socket interface via transcutaneous electrical neural stimulation	12 TF, 12 TT **Cause**: trauma/cancer/dysvascular disease/infection **Age**: 21–68 yrs (39.5 ± 13.3 yrs) **PE**: 3 mos.–31 yrs (8.1 ± 9.4 yrs) **TSA**: N/D	**FM**: Electrical neural stimulation **FD**: Transcutaneous electrodes **FS**: Concurrent **S/T**: Pressure transducers	Lab, N/D	Symmetry of weight distribution, single limb standing balance, step length symmetry, stance time symmetry	N/A	N/A	5 h to 6 h walking with/without feedback (10 min intervals per 20 min rest)	Both populations increased weight distribution symmetry, step length symmetry. Stance time symmetry and standing balance improved mainly for TF amputees.
[17] **Flowers et al. 1986** Develop and evaluate a BFB system to improve prosthetic weight-bearing and hip extension	5 TF **Cause**: N/D **Age**: 19–68 yrs **PE**: N/D **TSA**: N/D	**FM**: Auditory **FD**: Earphones or BFB speakers **FS**: Concurrent **S/T**: Load cell (or weight bearing transducers), goniometer	Lab, Overground	Weight bearing, hip extension angle, steps count	N/A	N/A	30 min to 1h sessions over 4 months (BFB device used during PT sessions)	Subjects with diminished awareness of their bodies and reduced strength benefited more from feedback. BFB improved hip extension and flexion at the beginning of stance phase
[92] **Clippinger et al. 1982** Enhance sensory feedback after lower limb amputation by providing electrical stimulation	13 LLA (5 Hip disarticulation, 7 TF, 1 bilateral TT) **Cause**: N/D **Age**: N/D **PE**: N/D **TSA**: 3 days–4 yrs	**FM**: Afferent sensory feedback **FD**: Surgically implanted electrodes **FS**: Concurrent **S/T**: Piezoelectric crystal, strain gauges	N/D, N/D	Weight bearing	N/A	N/A	3 h to 12 h of daily stimulation ranging from 8 months to 6 years	Implanted electrodes were tolerated by all patients without discomfort. Postoperative pain reduced and stump healing improved by stimulating the sciatic nerve
[93] **Gapsis et al. 1982** Evaluate a limb load monitor for controlling weight bearing of lower-limb amputees	20 LLA (n = 10 study, n = 10 control group) **Cause**: ND **Age**: 47–78 yrs (avg. 62.5 yrs) **PE**: N/D **TSA**: N/D	**FM**: Auditory **FD**: BFB buzzer **FS**: Concurrent **S/T**: Load sensitive transducer	Lab, Overground	Weight bearing (prosthetic limb load)	Total body weight	N/A	5 min for acclimation period, feedback system used during patient’s daily ambulation therapy	Control and study group reached goals. Study group reached goals twice as fast with feedback
[85] **Fernie et al. 1978** BFB device designed to promote knee extension at stance phase	19 TF **Cause**: ND **Age**: 46–84 yrs (avg. 70 yrs) **PE**: N/D **TSA**: N/D	**FM**: Auditory **FD**: BFB buzzer **FS**: Concurrent **S/T**: Foot and knee switch	N/D, N/D	Knee flexion/extension angle, steps count	Percentage of errors (i.e., bending the knee and loading the limb simultaneously)	N/A	3 weeks of training. PT involved at early BFB stages.	Feedback system encouraged knee flexion than knee extension. Audio signal too annoying to use.One participant showed a period of retention in the 3rd week of training
[94] **Zimnicki et al. 1976** BFB system developed for geriatric above-knee amputees to achieve an adequate knee extension during walking	13 TF **Cause**: N/D **Age**: 53–84 yrs (avg. 72 yrs) **PE**: N/D **TSA**: N/D	**FM**: Auditory **FD**: BFB buzzer **FS**: Concurrent **S/T**: Pylon switch	N/D, N/D	Knee flexion/extension angle, body weight pressure	N/A	N/A	5 progressive training stages over 5 or more sessions. PT involved to reinforce BFB training	BFB found to be more helpful for participants who had difficulty in following or concentrating on verbal instructions and for those one who appeared to understand but were enabled to elicit the appropriate motor responses
[95] **Warren et al. 1975** Evaluate the effectiveness of a BFB system in comparison to a Bathroom scale to improve weight-bearing	10 H **Cause**: N/A **Age**: 18–26 yrs **PE**: N/A **TSA**: N/A	**FM**: Auditory, Visual **FD**: BFB alarm **FS**: Concurrent **S/T**: Force plates, pressure sensitive insoles	Lab, Overground	Weight-bearing	N/A	N/A	Bathroom scale: two times - four steps on monitored leg. Three training levels with/without feedback (trying to reproduce target loading threshold)	BFB training was of limited value due to time lag between feedback and motor response

H: healthy subject; TT: transtibial (below-knee) amputation; TF: transfemoral (above-knee) amputation; PE: prosthetic experience; TSA: time since amputation; FM: feedback modality; FD: feedback device; FS: feedback strategy; S/T: sensors/transducers; SS: self-selected; PT: physiotherapist; N/A: not applicable; N/D: not described.

## Supplementary Materials

The following are available online https://www.mdpi.com/1424-8220/20/6/1628/s1. The PRISMA Checklist utilized to conduct the proposed systematic review is included as a supplementary material.
